# Case of Fistulous Communication between the Intestinum Ileum and Urinary Bladder, Simulating Stone in the Bladder

**Published:** 1845-01

**Authors:** W. C. Worthington

**Affiliations:** Surgeon to the Lowestoft Infirmary, and Fellow of the Royal Medical and Chirurgical Society


### MEDICO-CHIRURGICAL SOCIETY OF LONDON.

*Case of Fistulous Communication between the Intestinum Ileum and Urinary Bladder, simulating Stone in the bladder.* By W. C. Worthington, Esq., Surgeon to the Lowestoft Infirmary, and Fellow of the Royal Medical and Chirurgical Society. *Communicated by* James

Copland, M. D., F. R. S.—The patient, a female, sixty-five years of age, previously enjoying good health, began, four years ago, to suffer from pain in the right iliac region, the cause of which could not be satisfactorily traced. Having continued to experience this pain, symptoms indicating disturbance of the urinary organs commenced in November, 1842. Her pain was much aggravated. She had frequent and painful micturition; the urine was bloody, ropy, and highly offensive, and fragments of extraneous matter, the exact nature of which was not ascertained, were often found deposited from it. On sounding the bladder no calculus was felt, but there was a grating produced by moving the instrument, which led to its being supposed that some malignant ulceration had taken place in the coats of the bladder. The treatment consisted chiefly in giving anodynes to relieve the pain. The patient survived about four months, and died from an attack of diarrhœa. On examining the body after death, adhesions were observed between the convolutions of the intestines and the pelvic viscera. By further dissection it was found that a fold of the intestinum ileum was closely adherent to the fundus of the bladder, and that a communication existed between the two cavities by an ulcerated hole which extended through the coats of the intestine and the bladder, and was sufficiently large to admit the point of the index finger. On slitting open the bladder it was found partly filled with feculent matter and undigested food, such as currants, seeds, and other vegetable matter. The coats of the ileum near where the ulceration had taken place, were thickened and indurated, and the canal consequently strictured. The author concluded by quoting Dr. Copland's *Dictionary of Practical Medicine*, where similar cases are referred to with interesting observations on fistulous communications between the intestines and other viscera.

